# Percutaneous Coronary Intervention for the Anomalous Left Coronary Artery Originating from the Noncoronary Cusp

**DOI:** 10.1155/2016/2097174

**Published:** 2016-11-24

**Authors:** Toshiki Kuno, Yohei Numasawa, Toshiyuki Takahsashi

**Affiliations:** ^1^Department of Cardiology, Ashikaga Red Cross Hospital, Tochigi, Japan; ^2^Department of Cardiology, Koga Hospital, Koga, Japan

## Abstract

Percutaneous coronary intervention (PCI) for anomalous left coronary artery (LCA) originating from the noncoronary cusp (NCC) is challenging, as it poses difficulties with the engagement of the guiding catheter and the establishment of backup support. This report examines the case of a 69-year-old woman with unstable angina of anomalous LCA origin. The computed tomography showed a diffuse plaque in the middle of the left anterior descending (LAD) artery and an anomalous LCA originating from the NCC. After successful engagement of a straightened Judkins-Left diagnostic catheter, the angiography revealed a diffuse plaque in the middle of the LAD artery. We then engaged a Judkins-Right guiding catheter. Due to the weak backup support of the guiding catheter, we used another wire to stabilize it, and the stent was then implanted successfully. To our knowledge, this is the first case report of PCI for an anomalous LCA originating from the NCC.

## 1. Introduction

The anomalous origin of the left coronary artery (LCA) from the noncoronary cusp (NCC) is a rare congenital anomaly found in 0.008%–0.012% of coronary angiography cases [1, 2]. However, there have been few reports of percutaneous coronary intervention (PCI) for anomalous LCAs originating from the NCC. Due to the difficulty in correctly engaging the guiding catheter and in gaining backup support, the performance of PCI for an anomalous LCA originating from the NCC is challenging. We report a case of unstable angina of anomalous LCA origin in which the patient underwent a successful PCI.

## 2. Case Presentation

**Figure 1:**
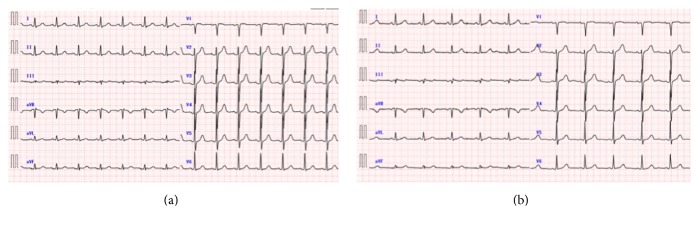
Electrocardiogram before (a) and after (b) the PCI.

**Figure 2:**
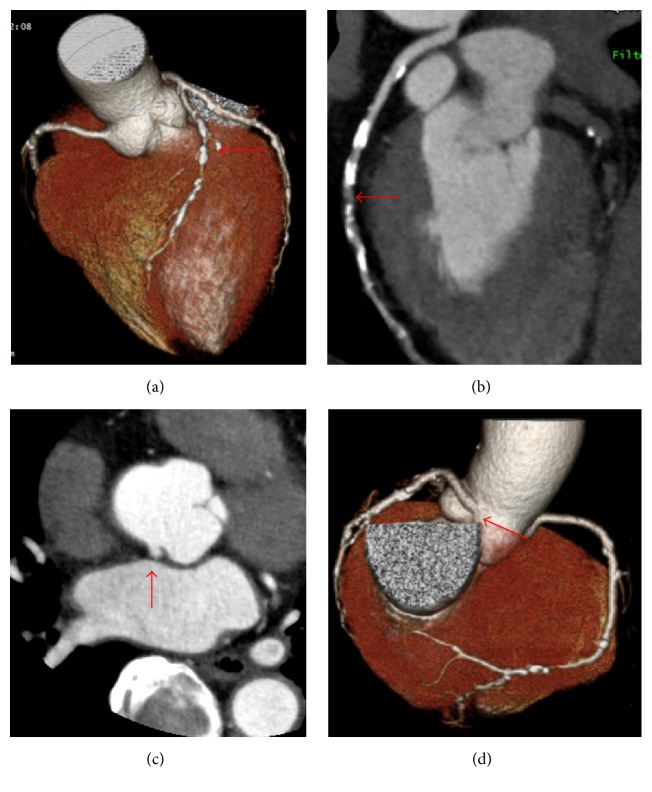
(a) CT (3D view) showing stenosis of the mid-LAD artery (indicated by the red arrow). (b) Multiplanar reconstruction view of the LAD artery (CT). The red arrow shows stenosis of the mid-LAD artery. (c) Red arrow showing arising ostium of the LCA (sectional view, CT). (d) Red arrow showing arising ostium of the LCA (3D view, CT).

**Figure 3:**
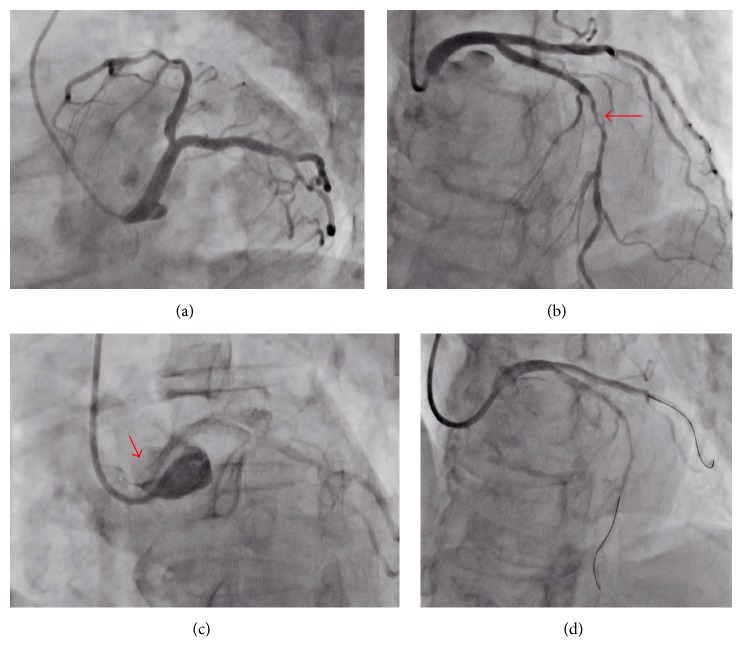
(a) Diagnostic coronary angiogram of the LCA (LAO/caudal view). (b) Diagnostic coronary angiogram of the LCA (LAO/cranial view). The arrow shows stenosis of the mid-LAD artery. (c) Cusp contrast injection with Amplatz-Left (LAO view). The arrow shows the ostium of the LCA. (d) Coronary angiogram with Judkins-Right guiding catheter with 2 wires.

**Figure 4:**
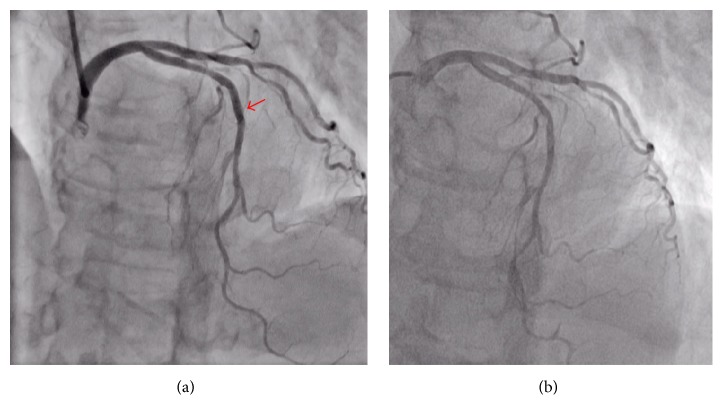
(a) Final coronary angiogram after stenting (red arrow). (b) Diagnostic coronary angiogram after 2 years of stenting.

A 69-year-old woman visited our hospital for chest pain at rest. She had a history of type 2 diabetes mellitus, hypertension, and dyslipidemia, and she had been smoking one pack of cigarettes per day. Her electrocardiogram did not show ischemic changes (Figure 1(a)), the transthoracic echocardiography revealed a normal systolic function, and her laboratory data (including the troponin T findings) were normal. Therefore, we ordered a coronary computed cosmography (CT). The CT showed a diffuse plaque in the middle of the left anterior descending (LAD) artery (Figures 2(a) and 2(b), red arrows) and an anomalous LCA originating from the NCC (Figures 2(c) and 2(d), red arrows). The patient was then admitted for a coronary angiography under suspicion of unstable angina. After her admission, we performed the coronary angiography by applying a 6-F sheath through a right trans-radial approach. Since we could not engage the 6-F Judkins-Left 3.5 Goodtec® (Goodman, Gifu, Japan) catheter smoothly because of the anomalous origin, we straightened it to engage it (Figure 3(a), Online Video 1 in Supplementary Material available online at http://dx.doi.org/10.1155/2016/2097174). After the catheter was engaged, the coronary angiogram showed 90% stenosis in the middle of the LAD artery (Figure 3(b), red arrow, Online Video 2). For engagement of the guiding catheter, we first chose a 6-F Amplatz-Left 0.75 Profit® (Goodman) to gain backup support. However, we were unable to engage it (Figure 3(c), Online Video 3). We then engaged a 6-F Judkins-Right 4.0 Profit (Goodman) catheter and proceeded with the PCI. After the engagement, we inserted a guide wire, Balance® (Abbott Vascular, Santa Clara, CA, USA), with a microcatheter, Mogule® (Goodman). However, due to the weak backup support, we could not proceed and advance the wire into the LAD artery. Therefore, we guided it to the left circumflex artery (LCX) with the Mogule. As we considered that we needed to enhance the backup support to the anomalous LCA, we replaced Balance with Grandslam® (Asahi Intec, Nagoya, Japan), which acted as an extra support wire. We then inserted the guiding catheter deeply and inserted Balance into the distal portion of the LAD artery (Figure 3(d), Online Video 4). We dilated the lesion with a 2.0 × 15 mm semicompliant balloon (Mini Trek® [Abbott Vascular]). After dilating the vessel, we delivered a 2.5 × 28 mm drug-eluting stent (Promus Element® [Boston Scientific, Boston, MA, USA]). Since the angiography showed that the stent was not entirely dilated, we dilated it with a 2.5 × 8 mm noncompliant balloon (Powered Lacrosse® [Goodman]). The patient's final coronary angiogram revealed successful stenting in the mid-LAD artery (Figure 4(a), Online Video 5). The patient was discharged without electrocardiogram changes (Figure 1(b)) and was asymptomatic at the 2-year follow-up point.

More recently, the patient developed chest pain. As the CT could not rule out stenosis of the stent's proximal edge, we performed a coronary angiogram. Since the right radial artery had not been suitable for smooth engagement of the diagnostic catheters 2 years prior, we attempted an angiogram through the left radial artery. The angiogram performed with an Amplatz-Left 1.0 diagnostic catheter showed the patent stent and no new lesions (Figure 4(b)). The patient was asymptomatic at the 3-month follow-up point.

## 3. Discussion

The anomalous origin of the left coronary artery (LCA) from the noncoronary cusp (NCC) is a rare congenital anomaly found in 0.008%–0.012% of coronary angiography cases [1, 2]. Although reports of percutaneous coronary intervention (PCI) for anomalous LCAs originating from the NCC are scarce, in our case, the performance of PCI for anomalous LCA originating from the NCC was challenging due to the difficulty in correctly engaging the guiding catheter and in gaining backup support. We were unable to engage an Amplatz-Left® 0.75 guiding catheter. Although an Ikari-Left® (Terumo, Tokyo, Japan) catheter would have been a good option to consider, as it is sometimes suitable for PCIs for right coronary lesions, including in cases of high take-off ostium [3], we eventually performed the PCI with a Judkins-Right®. However, because of the noncoaxial guiding catheter, the backup support for delivery of the stent was insufficient. We inserted a Grandslam in the LCX for enhanced support. Although the Guideliner® (Lifeline, Tokyo, Japan) was not yet available at the time, the mother-child technique would have been a valid option for smooth delivery of the stent [4].

After the follow-up period, the patient felt chest pain unrelated to in-stent restenosis. However, as in-stent restenosis could not be discarded without a coronary angiogram, we performed one by engaging an Amplatz-Left diagnostic catheter smoothly through the left radial artery. In this type of difficult case, a change in access route is an option for engagement of the catheter [5].

## 4. Conclusion

We report a case of unstable angina of anomalous LCA origin in which the patient underwent successful PCI.

## Supplementary Material

Online Video 1: Diagnostic coronary angiogram of the LCA (LAO/caudal view) Online Video 2: Diagnostic coronary angiogram of the LCA (LAO/cranial view). The angiogram shows stenosis of the mid left descending artery.Online Video 3: Cusp contrast injection with Amplatz-Left. The anigogram shows the ostium of left coronary artery.Online Video 4: Coronary angiogram with Judkins right guiding catheter with 2 wires Online Video 5: Final coronary angiogram after stenting

## Abbreviations

CT: Computed tomography
LAD: Left anterior descending
LCA: Left coronary artery
LAO: Left anterior oblique.
